# Molecular phylogenetics and evolutionary history of ariid catfishes revisited: a comprehensive sampling

**DOI:** 10.1186/1471-2148-9-175

**Published:** 2009-07-23

**Authors:** Ricardo Betancur-R

**Affiliations:** 1 Department of Biological Sciences, 331 Funchess Hall, Auburn University, Auburn, AL, 36849, USA; 2 Universidad Nacional de Colombia sede Caribe, CECIMAR/INVEMAR, Cerro de Punta Betín, Santa Marta, Colombia

## Abstract

**Background:**

Ariids or sea catfishes are one of the two otophysan fish families (out of about 67 families in four orders) that inhabit mainly marine and brackish waters (although some species occur strictly in fresh waters). The group includes over 150 species placed in ~29 genera and two subfamilies (Galeichthyinae and Ariinae). Despite their global distribution, ariids are largely restricted to the continental shelves due in part to their specialized reproductive behavior (i.e., oral incubation). Thus, among marine fishes, ariids offer an excellent opportunity for inferring historical biogeographic scenarios. Phylogenetic hypotheses available for ariids have focused on restricted geographic areas and comprehensive phylogenies are still missing. This study inferred phylogenetic hypotheses for 123 ariid species in 28 genera from different biogeographic provinces using both mitochondrial and nuclear sequences (up to ~4 kb).

**Results:**

While the topologies obtained support the monophyly of basal groups, up to ten genera validated in previous morphological studies were incongruent with the molecular topologies. New World ariines were recovered as paraphyletic and Old World ariines were grouped into a well-supported clade that was further divided into subclades mainly restricted to major Gondwanan landmasses. A general area cladogram derived from the area cladograms of ariines and three other fish groups was largely congruent with the geological area cladogram of Gondwana. Nonetheless, molecular clock estimations provided variable results on the timing of ariine diversification (~105-41 mya).

**Conclusion:**

This study provides the most comprehensive phylogeny of sea catfishes to date and highlights the need for re-assessment of their classification. While from a topological standpoint the evolutionary history of ariines is mostly congruent with vicariance associated with the sequence of events during Gondwanan fragmentation, ambiguous divergence time estimations hinders assessing the vicariant hypothesis on a temporal framework. Further examination of ariid fossils might provide the basis for more accurate inferences on the timing of ariine diversification.

## Background

The catfish order Siluriformes is a very diverse natural group that occurs primarily in freshwater. Catfishes are widespread and their distribution encompasses all continents, even Antarctica, as evidenced by Eocene-Oligocene fossils [1]. The order includes 36 extant families and over 3000 valid species plus an estimated ~1500 undescribed species [2,3]. Several morphological and molecular studies have addressed the relationships among catfish families [e.g., [4-6]] and recent evidence indicates that large basal clades are restricted to particular continental masses, suggesting a long history of intercontinental isolation [6]. Thus, catfishes offer an exceptional opportunity for studying evolutionary and biogeographic trends. The fossil record of Siluriformes is relatively well represented and includes material from every continent [7]. Although the earliest fossils date back to the Late Campanian-Early Maastrichtian (ca. 68-73 mya), molecular clocks predict a much older origin for Siluriformes (i.e., 175-130 mya [8-10]).

**Figure 1:**
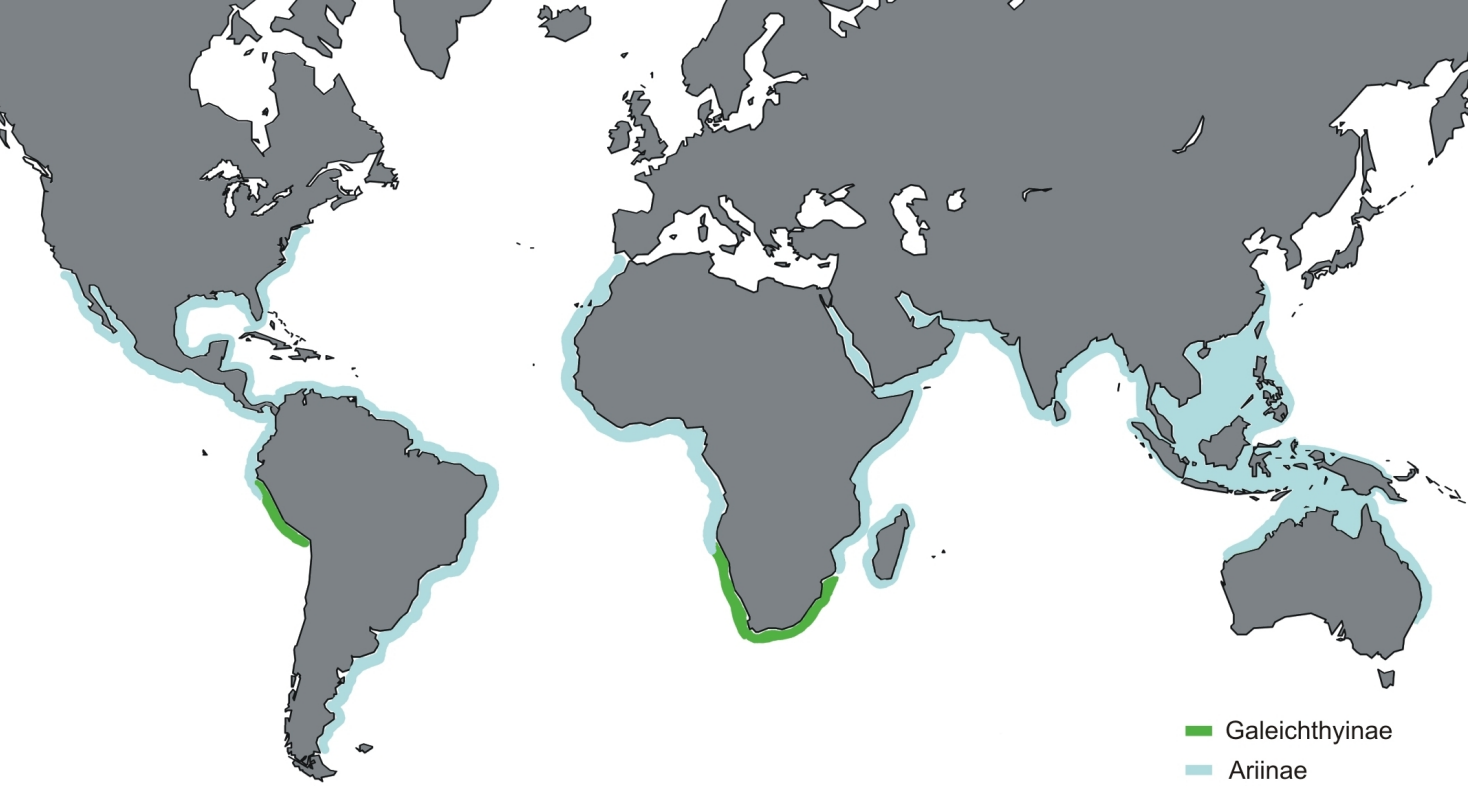
**Approximate distribution of ariids**. Some shaded areas represent extrapolated localities [after [19]].

While most catfishes inhabit freshwater, only two families are well represented in marine environments: the Plotosidae from the Indo-West Pacific and the Ariidae. The Ariidae, or sea catfishes, is the only siluriform group with a global distribution that includes over 150 species occurring in warm-temperate to tropical regions. Although most members of the family live in brackish and marine waters, several species occur in freshwater as well. Sea catfish distributions include the continental margins of the Eastern Pacific and the Western Atlantic (New World), the Eastern Atlantic (Western Africa), and the Indo-West Pacific (Eastern Africa, Madagascar, India-SE Asia, and Australia-New Guinea; Figure 1). Ariids play an important role in tropical fisheries, with many species having high economic value due to their large size, local abundance, and flesh quality. Some species have been recently listed as vulnerable on the IUCN red list [11].

Members of the Ariidae exhibit a specialized reproductive mode: male mouthbrooding of eggs and embryos. This condition is absent in nearly all other catfishes, the sole exception being the biparental mouthbrooding claroteid *Phyllonemus typus *[12]. Oral incubation means ariids have limited dispersal capabilities and subsequent high level of species endemism, which has ultimately resulted in continentally restricted distributions. In fact, ariids are absent from the Pacific plate [13], the nonmarginal portion of the Antilles, and other oceanic islands of recent volcanic origin [14]. Restriction of most species to the continental shelves make ariids an exceptional marine fish group to infer historical biogeography scenarios (although see [14] for evidence of recent transoceanic dispersal in *Galeichthys*). Such studies require a conceptual framework derived from robust phylogenetic hypotheses.

Recent interfamilial phylogenies based on morphological [15,16] and molecular [6] data have placed the Malagasy family Anchariidae as sister to the Ariidae and both families are ascribed in the superfamily Arioidea within the suborder Siluroidei [6]. The monophyly of the Ariidae has not been seriously questioned and is strongly supported on both molecular and morphological grounds [6,15,17]. The group is divided into two subfamilies, the monogeneric Galeichthyinae (four species) and the Ariinae (remaining taxa) [17]. Although the basal arioid clades are well defined, much controversy has arisen regarding the phylogeny and classification of ariid taxa, particularly within the diverse Ariinae.

**Figure 2:**
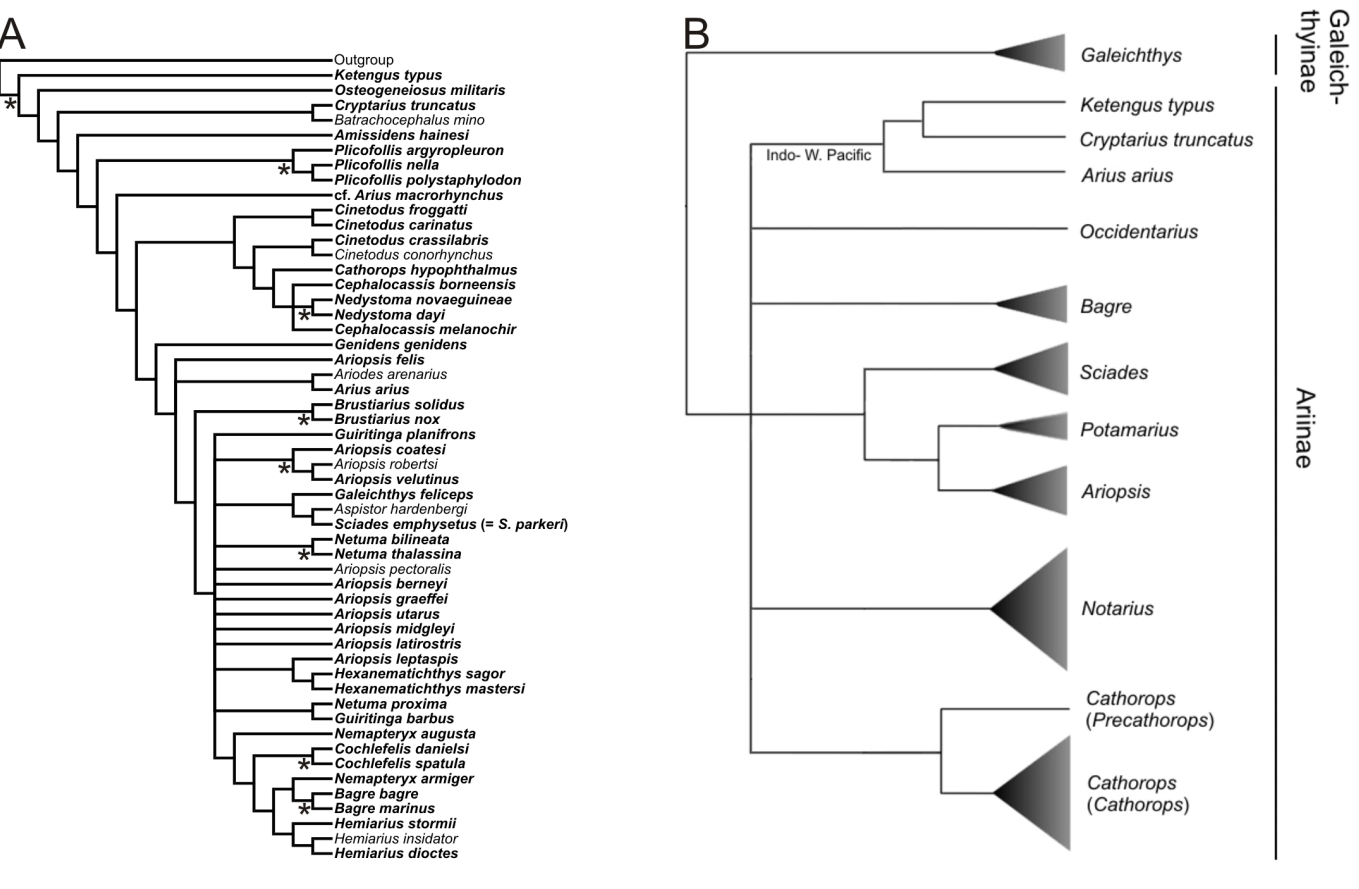
**Alternative hypotheses of relationships among ariid taxa**. (A) Kailola's [15] phylogeny on 45 Old World and eight New World ariid species based on 57 morphological characters. Taxa examined during this study are in bold; asterisks (*) indicate clades that are congruent with the topologies recovered (see Figure 3). (B) Betancur-R. et al.'s [19] phylogeny on 46 New World and three Old World ariid species. The summarized phylogeny is derived from trees obtained from mitochondrial (2842 bp), nuclear (978 bp), and morphological (55 characters) datasets. Both studies deal with different taxon-sampling schemes, and both topologies are highly incongruent regarding the position of *Galeichthys*, *Ketengus typus *and *Cryptarius truncatus*.

Recent studies that have attempted to elucidate relationships among ariids have mostly focused on taxa from restricted geographic areas and comprehensive phylogenies are still missing. Using anatomical data, Kailola [15] inferred relationships for 45 Old World and eight New World species (Figure 2A) and provided a revised classification accepting 23 genera. Based on combined evidence from mitochondrial and nuclear sequence data in addition to morphological characters [modified from [18]], Betancur-R. et al. [19] hypothesized relationships for 46 New World and three Old World species (Figure 2B) and provided a revised classification for New World taxa only, validating eight genera. Although the studies by Kailola and Betancur-R. et al. dealt with different taxon-sampling schemes, their resulting topologies are highly incongruent regarding the position of *Galeichthys *and the Indo-Pacific *Ketengus typus *and *Cryptarius truncatus *(Figure 2). In his unpublished doctoral dissertation, A. P. Marceniuk inferred phylogenetic relationships for 80 ariid species from different biogeographic provinces using morphological characters [20]. Following the results derived from this work, Marceniuk and Menezes [21] presented the most inclusive taxonomy for the Ariidae to date, recognizing 26 genera. More recently, Betancur-R. and Armbruster [14] inferred molecular phylogenies for the four species of galeichthyines showing that the Eastern Pacific species (*G. peruvianus*) is nested within a clade comprising the remaining three African species, and that the timing of intercontinental divergence occurred from Mid to Late Miocene, likely implying transoceanic dispersal. As will be discussed below, not only the molecular and the anatomical hypotheses differ markedly, but also the two morphological classifications show considerable disagreement.

This study expands previous molecular phylogenies on New World ariids and galeichthyines using mitochondrial sequences (*cytochrome b*, *ATP synthase 8/6*, *12S*, and *16S*; ~3 kb) and a nuclear marker (*rag2*, ~1 kb) to cover a wide spectrum of taxa from different biogeographic provinces. In addition to the 63 ariid species examined previously [14,19], new molecular data was obtained for 60 other species. These data were utilized for three main purposes: (1) hypothesize ariid relationships via maximum parsimony (MP), maximum likelihood (ML), and Bayesian inference (BI) reconstruction criteria; (2) discuss morphological phylogenies and current classifications in the light of molecular phylogenies; and (3) infer historical biogeography scenarios for the Ariinae using cladistic and chronological methods. This study provides the most inclusive phylogeny of ariid taxa to date and a resource for future classifications and other comparative studies in the family.

## Results and discussion

### Dataset attributes

**Figure 3:**
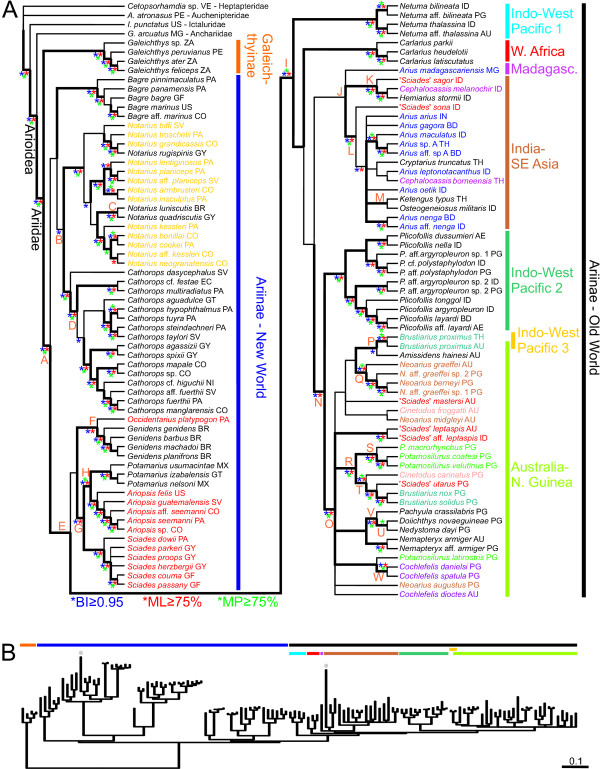
**BI phylogeny of 124 arioid species derived from the mitochondrial dataset (2866 bp)**. Fifty percent majority rule consensus on ~2.15 × 105 post-burn-in trees (mean lnL-52160). (A) cladogram; thicker branches indicate clades that are congruent with MP and ML (Garli and RAxML) analyses. Asterisks (*) designate clade support (see also Additional file 2); capital letters indicate nodes referred in text and Additional file 2 (symbols and letters always on left of nodes); vertical bars indicate subfamilial divisions and distribution of major ariine groups. Generic placement for New World and Old World ariines follows Betancur-R. et al. [19] and Marceniuk and Menezes [21], respectively. Colored taxa indicate non-monophyletic genera validated by Marceniuk and Menezes (yellow and red taxa correspond to *Notarius *and *Sciades *sensu [21], respectively). Two letter country codes follow ISO-3166. (B) phylogram (Ariidae only) elucidating the short internodes at the base of the Ariinae and the rate variation across lineages (taxon arrangement follows the same order in both figures). Gray dots indicate long branches in *N. lentiginosus *(left) and *H. sagor *(right).

All sequences obtained during this and previous studies [6,14,19,22-33] are available from GenBank under accession numbers listed in Additional file 1. The final alignment of the mitochondrial protein coding genes included 1095 bp for partial *cyt b *and 842 bp for *ATPase 8/6 *[see details in [19]]. Indels were only observed in *ATPase 8*, where *Cinetodus carinatus*, ten species of *Notarius *(including *N. lentiginosus*), and '*Sciades*'*sagor *lacked a codon 108, 111, and 141 bases downstream of the start codon, respectively. For *ATPase 6*, three initiation codons were observed: UUG in *S. sagor *and the species of the subgenus *Cathorops*, GUG in *Notarius planiceps*, *Notarius aff. planiceps*, and *N. lentiginosus*, and the typical AUG in the remaining taxa. Likewise, *N. lentiginosus *presented UUG instead of AUG in *ATPase 8*. The start codons GUG and UUG have been shown to be less efficient variants of AUG in some genes [34]. In addition to the differences observed in *Notarius lentiginosus *and *S. sagor *at the amino acid level, both species revealed the highest variation in substitution rates of mtDNA among ariid taxa (Figure 3B). It is noteworthy that no ambiguous chromatogram readings, stop codons or frameshifts were observed in these two species, suggesting that these sequences are likely not pseudogenes.

Partial ribosomal 12S and 16S sequences ranged in size from 385 to 394 bp and 556 to 571 bp, with final alignment lengths of 406 and 595 bp, respectively. In the final alignments, 18 sites of 12S and 54 sites of 16S were excluded due to ambiguous positional homology. The mitochondrial dataset assembled included 129 terminal taxa (124 arioid species including one anchariid, four galeichthyines, and 119 ariines) and 2866 sites. The dataset had missing data for *Notarius luniscutis *in *ATPase 8/6 *(due to amplification failure) and for *Cathorops manglarensis *(65 bp at 3' end) and *N. lentiginosus *(35 bp at 3' end) in *16S *(due to polymerase slippage producing noisy sequences). Many other taxa also had missing data on sequence ends due to noisy chromatogram reads. Considering only arioid taxa, the mitochondrial alignment contained 1273 (44.4%) variable sites and 1097 (38.3%) parsimony-informative sites, respectively.

The nuclear *rag2 *gene has been shown to have low variability within the Ariidae [19], thus nuclear sequences were obtained only from major lineages (as indicated by the mitochondrial topologies) representing all biogeographic provinces and sampled genera (73 species). Due to amplicon-length variation from different primer combinations or noisy chromatogram endings, in the nuclear *rag2 *alignment not all ingroup taxa had the same sequence length, varying from 837 to 978 bp; outgroups had 720 bp [obtained from [6,23]]. No insertions or deletions were observed in the *rag2 *dataset. The number of variable and parsimony-informative sites for the ingroup in the *rag2 *dataset were 174 (17.8%) and 67 (6.9%), respectively. In addition to the mitochondrial and nuclear partitions, a combined dataset including 73 common taxa was assembled (73 species, 3844 sites, 1525 variable sites, 1157 parsimony-informative sites).

### Phylogenetic inference

Optimality results obtained under different analyses and model testing on the three data partitions are summarized in Table 1. Among the four reconstruction methods conducted (MP, ML-Garli, ML-RAxML, BI) on the mitochondrial dataset, BI analysis resulted in the least resolved tree. The BI consensus is shown in Figure 3; congruent nodes recovered under other methods are indicated by thicker lines. As found in previous molecular studies [6,19], the superfamily Arioidea sensu Sullivan et al. was recovered as monophyletic, with the Anchariidae sister to the Ariidae (all nodes strongly supported). The subfamilial divisions within the Ariidae (Galeichthyinae and Ariinae) were fully congruent among different methods and well supported. The relationships within the Galeichthyinae and among New World ariine genera are basically identical to those reported in previous molecular studies [14,19] (Figure 2B). The new finding is that the genus *Genidens *from the Western Atlantic (previously not examined) was monophyletic and sister to the monotypic *Occidentarius *from the Eastern Pacific (node F, Figure 3).

**Table 1:** Summary of initial conditions and results obtained in phylogenetic reconstructions and model testing.

**Analysis**	**Mitochondrial**	**Nuclear**	**Combined**
	129 taxa, 2866 bp	73 taxa, 978 bp	73 taxa, 3844 bp
**MP**			
RA replicates	100	5	100
Bootstrap replicates	1000 (10 RA rep.)	_	1000 (10 RA rep.)
Optimal trees retained	8	>5 × 10^4^	36
Optimal tree score (steps)	11208	387	9002
Consistency index	0.188	0.749	0.25
Consensus type	strict	50% majority rule	90% majority rule
**Initial model (ML and BI)**			
Akaike information criterion	GTR+I+Γ	HKY+I+Γ	GTR+I+Γ
Number of substitution rate parameters	6	2	6
**ML – Garli**			
Search replicates	10	5	10
Automatic termination* (generations)	1 × 10^4^	1 × 10^4^	1 × 10^4^
Optimal tree score (lnL)	-52003.50	-3791.51	-43666.78
Bootstrap	_	_	_
**ML – RAxML**			
Search replicates	10	5	5
Partitions	none	none	none
Optimal tree score (lnL)	-52145.37	-3790.72	-43726.10
Bootstrap replicates**	150	400	200
**BI**			
Search replicates	3	3	3
Partitions	none	none	none
Generations	1 × 10^7^	6 × 10^6^	6 × 10^6^
Burn-in	2.5 × 10^6^	1.5 × 10^6^	1.5 × 10^6^
Post-burn-in trees (combined searches)	~2.15 × 10^5^	~1.35 × 10^5^	~1.35 × 10^5^
Mean lnL	-52160	-4119.14	-43760
Efective Sample Size (all parameters)	>200	>200	>200
Consensus type	50% majority rule	50% majority rule	50% majority rule

RA, random addition

*genthreshfortopoterm command

**rapid bootstrapping algorithm via automatic termination

Delimitations of New World genera [sensu [19]] were congruent with the mitochondrial topologies; however, seven Old Word genera [sensu [21]] were found to be non-monophyletic (Figure 3, colored taxa; see below). In general, the relationships among basal ariine taxa were poorly resolved or supported, with short internodes (Figure 3B). While New World taxa were not monophyletic in any of the analyses, Old World ariines were grouped into a well-supported clade (node I, Figure 3). The basal nodes linking some New World genera (e.g., *Occidentarius*, *Genidens*, *Potamarius*, *Ariopsis*, and *Sciades*) to the Old World clade were often incongruent among different reconstruction criteria and poorly supported (node E, Figure 3). Within the Old World clade, taxa were generally grouped into well-defined biogeographic assemblages (Figure 3, colored bars). Overall inshore species conform to major Gondwanan provinces (Africa, Madagascar, India-SE Asia, and Australia-New Guinea or Sahul). Delimitations and nodal supports for such groups were high, except for the India-SE Asia assemblage that was often recovered as non-monophyletic with the Madagascar species (*Arius madagascariensis*) nested within. Offshore taxa found widely distributed along Indo-West Pacific shelves fell into three categories: the genera *Netuma *(Indo-West Pacific 1) and *Plicofollis *(Indo-West Pacific 2), and *Brustiarius proximus *(Indo-West Pacific 3, nested within the Australia-New Guinea clade; Figure 3). Resolution among Old World biogeographic clades was poor, except for the sister-relationship between Australia-New Guinea and *Plicofollis *(node N, Figure 3), which was fully congruent and well supported.

The India-SE group comprised two well-supported basal clades, with one including '*Sciades*' *sagor*, *Cephalocassis melanochir*, and *Hemiarius stormii *(node K, Figure 3) and other grouping the remaining taxa (node L, Figure 3). The latter clade was largely unresolved. The relationships within *Netuma*, *Carlarius*, and *Plicofollis *were entirely resolved with high nodal support. There was poor resolution within the Australia-New Guinea assemblage (node O, Figure 3), except for the following well supported-clades: *Brustiarius proximus + Amissidens hainesi *(node P, Figure 3); *Neoarius *(in part; node Q, Figure 3); node R (Figure 3): node S (*Potamosilurus*, in part) + node T (*Cinetodus carinatus*, ('*Sciades*' *utarus*, (*B. nox*, *B. solidus*))); *Pachyula crassilabris *+ *Doiichthys novaeguineae *+ *Nedystoma dayi *(node V, Figure 3); and *Cochlefelis *(in part; node W, Figure 3).

The congruences between methods and data partitions (mitochondrial, nuclear, and combined) are summarized in Additional file 2. There was strong phylogenetic signal in the mitochondrial dataset, often yielding well-resolved and well-supported nodes. The weak signal and the small number of characters in the nuclear *rag2 *dataset (67 parsimony-informative sites) resulted in the greatest number of incongruent nodes (Additional file 2). Although using a more reduced taxon-sampling scheme, the combined partition yielded highly congruent topologies as compared to the mitochondrial topologies. The major difference is that the combined scheme recovered the India-SE Asia group as monophyletic in all topologies (only MP and ML-Garli in the mitochondrial partition), but failed to place the Madagascar species sister to the India-SE Asia clade. Incongruence between mitochondrial and nuclear datasets may be alternatively due to gene-tree/species-tree conflicts [35,36]. Thus, given this situation, further exploration of ariid phylogeny should emphasize the inclusion of additional, independent nuclear markers.

The lack of resolution and short internodes evidenced among basal ariine lineages is consistent with the reconstructions conducted on the mitochondrial, nuclear, and combined partitions. Given that mitochondrial and nuclear markers contain different levels of signal [19], this pattern may be the result of rapid ariine radiations; however, further analyses are required to test this hypothesis [see [37]].

### Morphological hypotheses, classifications, and alpha taxonomy

The morphological phylogeny hypothesized by Kailola (Figure 2A) differs substantially from molecular topologies (Figure 3; Table 2). Of the 34 possible nodes common to both datasets (mitochondrial partition), only eight clades are congruent (Figure 2A, asterisks). Further MP and ML reconstructions constraining Kailola's topology into the mitochondrial dataset yielded scores significantly worse than those obtained under unconstrained searches (Templeton and SH tests; Table 2). Some of the most striking differences between the two hypotheses are the nested position of *Galeichthys*, the basal position and non-monophyly of Old World taxa, and the polyphyly of *Genidens *in Kailola's cladogram. Thus, Kailola's topology does not support the Galeichthyinae and Ariinae as basal subfamilial divisions. Remarkably, unlike the molecular topologies, there are no distinguishable biogeographic patterns in the morphological hypothesis as ariine taxa from different regions appear randomly scattered across the tree. Based on Hennig's [38] principle of reciprocal illumination, congruence between biogeography and phylogeny provides a logical framework to favor the molecular over the morphological hypothesis.

**Table 2:** Incongruence between phylogeny and classifications derived from morphological studies and the molecular evidence.

	**# of taxa in constrained clade**	**SH** **(*p *value)**	**Templeton** **(*p *value)**
**Kailola (2004)**			
Phylogeny	48 (34 constrained nodes)	**0.000**	** < 0.0001**
*Ariopsis*	20	**0.000**	** < 0.0001**
*Arius*	7	0.411	0.72–0.88
*Aspistor*	11	**0.000**	** < 0.0001**
*Cephalocassis*	2	**0.000**	** < 0.0001**
*Cinetodus*	3	**0.000**	** < 0.0001**
*Hemiarius*	4	**0.000**	** < 0.0001**
*Hexanematichthys*	2	**0.027**	** < 0.01**
*Nemapteryx*	5	**0.000**	** < 0.001**
*Netuma*	5	**0.018**	** < 0.01**
*Sciades*	8	**0.000**	** < 0.0001**
**Marceniuk and Menezes (2007)**			
*Arius*	10	**0.004**	** < 0.04**
*Arius *excluding *A. madagascariensis*	9	0.341	0.59–0.77
*Brustiarius*	3	**0.000**	** < 0.0001**
*Cephalocassis*	2	**0.000**	** < 0.0001**
*Cinetodus*	2	0.153	< 0.08*
*Cochlefelis*	3	0.355	0.31–0.41
*Neoarius*	6	0.132	0.18–0.25
*Notarius*	13	**0.013**	** < 0.03**
*Potamosilurus*	4	**0.014**	0.09–0.13
*Sciades*	18	**0.000**	** < 0.0001**

Results obtained with Templeton and Shimodaira-Hasegawa (SH) tests of topology congruence between trees constrained under the morphological hypotheses and unconstrained trees (significant *p *values in bold). Hypothesis testing was performed on Kailola's phylogeny [15] (see Figure 2A) and those genera defined by Kailola [15] and Marceniuk and Menezes [21] that were recovered as non-monophyletic (see also Figure 3). For generic comparisons, only one node was constrained. Taxa in constrained clade include common species only; however, for some comparisons the number of taxa with enforced monophyly is greater than the number of taxa assigned to a particular genus in previous studies due to the greater number of species recognized here (e.g., *affinis *entities).

*In 52 out of 56 comparisons *p *value < 0.05

Conflicts in phylogenetic hypotheses also have profound implications on the classification. Despite their economic importance, the taxonomy of the Ariidae has for a long time remained in a chaotic state and is probably the most unresolved among catfish families [3]. This has caused a vast nomenclatural instability with species commonly jumping from one genus to another. Even the three recent studies that have addressed the classification of ariids reveal considerable disagreement [15,19,21]. The results presented here also differ from previous classifications in several aspects.

Despite the more restricted taxon sampling, the previous classification of New World taxa based on combined molecular and morphological characters [19] is fully congruent with current topologies. This study also validates the status and confirms monophyly of the New World genus *Genidens *[not included in [19]]. The major conflicts concern the morphology-based taxonomies as ten genera recognized by Kailola [15] and nine by Marceniuk and Menezes [21] (Figure 3A) were not monophyletic in any of the reconstructions conducted on the mitochondrial partition (broadest taxon sampling). Furthermore, Templeton and SH tests constraining each non-monophyletic genus into the mitochondrial dataset identified strong departures from congruence in 16 (nine in [15]; seven in [21]) out of the 19 comparisons (Table 2). Similar results were also obtained on reconstructions performed on single-gene partitions (results not shown).

**Figure 4:**
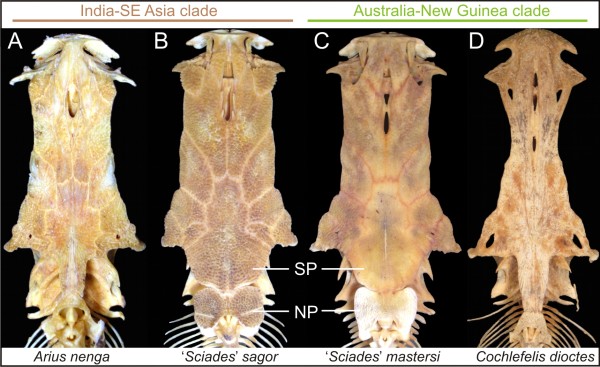
**A remarkable example of morphological convergence**. The genus *Hexanematichthys *sensu Kailola [15] includes two species (*'Sciades' sagor *and *S. mastersi*) and is defined by the presence of a broad and depressed head, a short and broad supraoccipital process (SP) and a large butterfly-shaped nuchal plate (NP), among other features. While the neurocrania of *S. sagor *and *S. mastersi *are most similar in this sample, the molecular evidence suggests that they are more closely related to *Arius nenga *(India-SE Asia clade) and *Cochlefelis dioctes *(Australia-New Guinea clade), respectively (see Figure 3). Also, Templeton and SH tests reject monophyly of *Hexanematichthys *(see Table 2). (A), AUM 46280, 87 mm cranial length (CL); (B), AUM 50242, 131 mm CL; (C), AUM 47562, 117 mm CL; (D), AUM 47507, 170 mm CL.

The definition and delimitation of most conflicting genera not only differ markedly among the two anatomical studies, but also have no logical biogeographical circumscriptions. For instance, the genera *Ariopsis*, *Aspistor*, and *Hemiarius *sensu Kailola and *Sciades *sensu Marceniuk and Menezes are ubiquitous and comprise numerous unrelated species/clades (>16 species in *Ariopsis *and *Sciades*). Likewise, *Hexanematichthys *and *Nemapteryx *sensu Kailola both include species that belong either in the India-SE Asia or the Australia-New Guinea clades. Interestingly, *Hexanematichthys *sensu Kailola provides a remarkable example of morphological convergence (Figure 4). As defined by Kailola, *Hexanematichthys *includes two species ('*Sciades*'*sagor *from India-SE Asia and *S. mastersi *from Australia-New Guinea) and is diagnosed by the presence of a broad and depressed head, a short and broad supraoccipital process, a large butterfly-shaped nuchal plate, and a dark peritoneum, among other features [15] (some of these characteristics also present in the Neotropical *Sciades *and some *Notarius *[21,39]). While there is an extraordinary similarity between the neurocrania (and other features) of *S. sagor *and *S. mastersi *(Figure 4) and the morphological phylogeny places the two species as sister taxa (Figure 2A), the monophyly of *Hexanematichthys *is not supported by the molecular data (Figure 3; Table 2). *Cephalocassis *(from SE Asia) and *Cinetodus *(from Australia-New Guinea) are additional examples of genera that are reasonably well established morphologically (although their delimitations vary in Kailola [15] and Marceniuk [21]), but incongruent on a molecular basis (Table 2). Noteworthy, *Cephalocassis melanochir *and *C. borneensis *are two of the eight purely freshwater species found in SE Asia. Both species share several features, including a fenestra between the supraoccipital, the pterotic, and the sphenotic, unique among ariids [[15,21]: Fig. thirtyseven]. Their morphological similarity may be the result of convergence associated with the transitions between marine and freshwaters.

The genus *Arius *has been one of the major problems concerning ariid systematics. As traditionally recognized, *Arius *is the largest and most widespread genus in the family [e.g., [40,41]]. All recent studies [15,19,21] concur that the genus includes considerably fewer species than was previously accepted (< 25) and, unlike prior classifications, no New World species are currently placed in *Arius*. Although *Arius *sensu Kailola was not recovered as monophyletic, it is restricted to the India-SE Asia province and the topology with enforced monophyly was not rejected by the mitochondrial data. Similarly, the monophyly of *Arius *sensu Marceniuk and Menezes is not rejected by the topological tests, but only if the Malagasy species (*A. madagascariensis*) is excluded (Table 2). From a phylogenetic standpoint, the present results also suggest that the genera *Netuma *and *Sciades *sensu Kailola and *Brustiarius*, *Cochlefelis*, *Neoarius*, and *Potamosilurus *sensu Marceniuk and Menezes each include only a few species incorrectly assigned to them.

In the light of the classification adopted for this study (see Materials and Methods), *Notarius *[sensu [19]], *Cathorops*, *Arius*, and *Plicofollis *are among the most species-rich genera in the family. Delimitation of the Neotropical *Notarius *sensu Marceniuk and Menezes is similar to that of Betancur-R. et al., except that Marceniuk and Menezes placed *N. rugispinis *and *N. phrygiatus *in their new genus *Amphiarius*, and *N. luniscutis *and *N. quadriscutis *in *Aspistor*. Validating *Amphiarius *and *Aspistor *would render *Notarius *paraphyletic (although in some analyses *Aspistor *was recovered as sister to all other *Notarius*; see Table 2). *Aspistor *was also recognized by Kailola but with a different circumscription, including seven species of *Notarius *sensu Betancur-R. plus *Hemiarius hardenbergi *(from Australia-New Guinea) and *Occidentarius platypogon*. The monophyly of *Notarius *sensu Marceniuk and *Aspistor *sensu Kailola are not supported by the molecular evidence (Table 2). A consensus solution for *Notarius *would be providing subgeneric assignments.

Molecular evidence also brings new perspectives at the alpha taxonomy level. While recent taxonomic check lists estimate that the number of valid ariid species range from 125 to 136 ([15,21]; plus five more recent additions [42-44]), there are 53 species designated as inquirendae in the family (i.e., uncertain validity), the greatest number within the order Siluriformes [3,39]. The major difficulties in undertaking a comprehensive taxonomic study of the Ariidae are the overall similarity in external morphology, the widespread distribution of the group coupled with the high degree of species endemism, and the poor representation of species diversity in museums [see also [21]]. Although estimating the total number of valid species is a challenging task, examination of wide variety of taxa on both mitochondrial and/or morphological grounds enabled identification of 20 putative undescribed/unrecognized species (referred as *affinis *or sp.; see Materials and Methods). The alpha taxonomy of the Ariidae is in need of revision.

Despite the recent efforts to clarify ariid taxonomy [15,19,21], these results show that it is still far from settled. This instability prevents an adequate management of the fisheries, making ariids a difficult group for conservational purposes. A total evidence approach based on combined morphological and molecular data would provide a suitable framework to redefine genera and to reassess the classification of the Ariidae. This issue will be addressed elsewhere.

### Historical biogeography of the Ariinae

#### -The evolutionary history of ariines from a topological perspective

Presently, ariid distribution encompasses the New World, Africa, Madagascar, India-SE Asia, and the Sahul continent (Figure 1); however, as evidenced by the fossil record, the group also ranged into Europe until the Late Miocene. The current poor representation of catfishes in Europe has been attributed to extinctions caused by Pleistocene glaciations [45]. While galeichthyines are restricted to subtropical waters in southern Africa and southwestern South America, sea catfishes owe their broad distribution to ariines. The disjunct biogeography of galeichthyines was recently addressed [14], thus this section focuses on ariines only.

**Figure 5:**
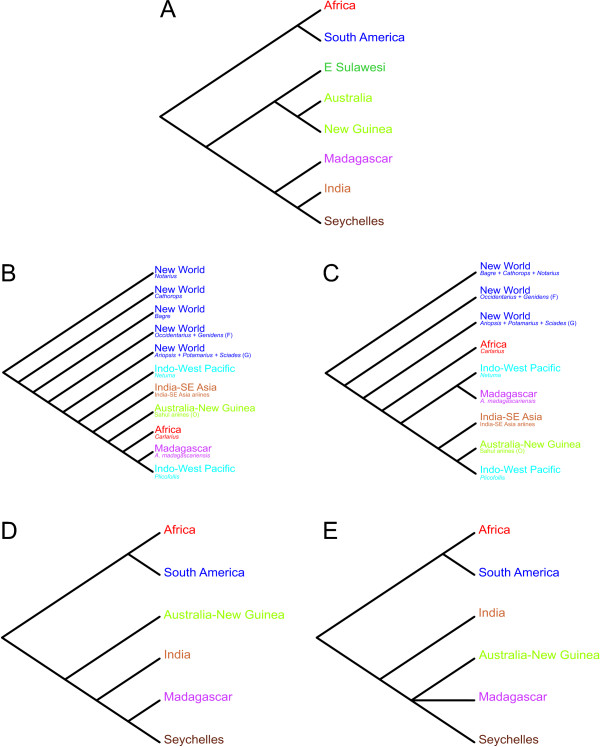
**Area cladograms**. (A) Geological area cladogram of Gondwanan progression (summarized by [48] based on [55] and [56]). Ariine area cladograms based on MP (B) and ML (C) topologies estimated on the combined dataset (see details in Table 1, Additional file 2). (D) Sparks and Smith [48] general area cladogram derived from the area cladograms of cichlids, aplocheiloid killifishes, and rainbowfishes (for particular area cladograms see [[48]: Fig. four]). (E) General area cladogram derived from the component analysis of the four fish groups using either MP (strict consensus of three optimal trees, minimal value = 51) or ML (strict consensus of three optimal trees, minimal value = 32) topologies. Widespread *Netuma *and *Plicofollis *were handled under assumption 2 [76-78,81] by arbitrarily removing all but one area from their distributions (areas used: India-SE Asia for *Netuma*, Australia-New Guinea for *Plicofollis *[79,80]). Letters in parentheses refer to nodes in Figure 3 and Additional file 2.

The presence of ariines on major landmasses of the southern hemisphere suggests (*a priori*) a Gondwanan vicariance [but see [46,47]]. To further test this hypothesis, the biogeography of the Ariinae was approached under topological and chronological frameworks. Area cladograms for Gondwana (based on geological data) and ariine taxa (based on combined evidence trees), and general area cladograms derived from various fish groups are shown in Figure 5. Different reconstruction methods yielded different topologies (Additional file 2), thus ariine area cladograms were derived from MP (Figure 5B) and ML (Figure 5C) trees (BI and ML topologies are congruent/combinable). The reconstructions indicate that New World ariines are basal and paraphyletic while Old World taxa form a nested clade further subdivided into groups endemic to major areas (i.e., Africa, Madagascar, India-SE Asia and Australia-New Guinea).

In addition to the marked biogeographic associations, the Old World clade includes two subclades with widespread Indo-West Pacific distributions (*Netuma *and *Plicofollis*). Likewise, although *Brustiarius proximus *is nested within the Australia-New Guinea clade, its range extends into SE Asia (reported here for the first time). DIVA analyses indicate that the presence of *B. proximus *in SE Asia is most parsimoniously the result of dispersal from Australia-New Guinea. Thus, for component analyses, *B. proximus *is treated as a Sahul taxon. Inferring the biogeographic history of the widespread *Netuma *and *Plicofollis *is less straightforward because of ambiguous relationships among major Old World clades (Figs. 5B, C). According to DIVA optimizations on the mitochondrial dataset (broadest taxon sampling), the distribution of the common *Plicofollis *ancestor was either India-SE Asia or the composite India-SE Asia/Australia-New Guinea, whereas the ancestral distribution of *Netuma *was India-SE Asia/Australia-New Guinea.

Overall, the major differences between ariine (Figs. 5B, C) and geological area cladograms (Figure 5A) are the absence of ariine endemics from Sulawesi and the Seychelles and the lack of a close relationship between New World and African taxa. However, further analyses constraining the monophyly of the New World node G (*Potamarius + Ariopsis + Sciades*; see Figure 3) plus the African genus *Carlarius*, yielded scores similar to those obtained under unconstrained searches (SH test *p *value = 0.35; Templeton test *p *value = 0. 06–0.29). Similarly, while the placement of Australia-New Guinea, India-SE Asia, and Madagascar is variable among different reconstructions (Figs. 5B, C) and the predicted area relationship based on the Gondwanan model was not recovered (but see Garli ML reconstructions on the mitochondrial dataset; Additional file 2), additional analyses enforcing the Australia-New Guinea + (India-SE Asia + Madagascar) topology are not statistically rejected by the data (SH test *p *value = 0.472; Templeton test *p *value = 0.35–0.54; note that *Netuma *and *Plicofollis *were excluded from the analysis because their widespread distributions).

Component analyses on both MP and ML topologies derived identical general area cladograms (Figure 5E). The general fish area cladogram based on ariines, cichlids, aplocheiloid killifishes, and rainbowfishes (Figure 5E) is largely congruent with Sparks and Smith's [48] general area cladogram derived from the latter three groups only (Figure 5D) and the geological area cladogram (Figure 5A). The main difference is the truncated placement of Australia-New Guinea and India-SE Asia (Figure 5).

Although ariines are predominantly marine and therefore potentially capable of dispersing along contiguous intercontinental shelves (e.g., *Netuma*, *Plicofollis*, *Brustiarius proximus*), the fact that most taxa with limited dispersal capabilities were grouped into major Gondwanan clades and that relationships among these clades largely coincide (or at least are not significantly incongruent) with the geological history of the super continent, suggest vicariance via continental drift. While restricted distributions are the generalized condition within the Ariinae, widespread Indo-West Pacific taxa are derived from three independent acquisitions (Figure 3). If dispersal is historically the major force driving ariine biogeography, no provincial associations would be observed. As pointed out by Sparks and Smith [49] for cichlids, a close relationship between African and Malagasy taxa across the Mozambique canal is expected under a dispersalist model. However, the reconstructions presented here offer no evidence for Afro-Malagasy clades (although the two ariine species from Eastern Africa were not examined). Similarly, the sharp faunal division across Wallace's line (dividing SE-Asia and Australia-New Guinea plates) of most (i.e., non-widespread) ariine taxa disfavors dispersal acting as a homogenizing force. The collision of the India subcontinent into Asia 43 mya [50,51] permitted faunal dispersal to SE Asia but not to the Australia-New Guinea plate due to deep oceanic barriers preventing faunal exchange across Wallace's line (except for the three Indo-Pacific taxa found in SE Asia and Australia-New Guinea). Remarkably, with the exception being the basal node linking New and Old World ariines (e.g., node E, Figure 3), the topologies do not show any transpacific or transatlantic nested clades within the Ariinae, supporting the idea that sea catfishes have limited transoceanic dispersal capability (but see [14] for evidence of recent dispersal from Africa to South America in the Galeichthyinae). As Sparks and Smith [49] argued, not only freshwater fishes in the primary division but also continentally restricted marine groups may be of great value for inferring ancient land connections.

The presence of ariines in non-Gondwanan areas such as Mesoamerica/North America and Europe (until Late Miocene) was probably the result of contiguous intercontinental marine dispersal from South America and perhaps Africa, respectively. In support for this scenario, DIVA analyses derived from the mitochondrial dataset (broadest taxon sampling) indicates South America as center of origin for New World taxa. Given that extant ariids are absent from Europe (but see records of the African *Carlarius parkii *in the Mediterranean [52]) further morphological studies including European fossils are required to test dispersal into this continent.

#### -The timing of ariine diversification in the context of Gondwanan vicariance

Recent studies have shown a variety of floral and faunal taxa whose disjunct distributions in the southern hemisphere, previously associated with Gondwanan vicariance, likely represent instances of transoceanic dispersal. Particularly, molecular dating techniques have revealed divergence times that are too young to be explained by continental-drift vicariance. Remarkable examples of recent transoceanic dispersal include geckos, monkeys, lemurs, carnivores, chameleons, frogs, insects, and several angiosperm families, among others [reviewed in [46]]. Contemporary advocates of dispersal even argue that vicariance should be revaluated as the most likely a priori assumption in biogeography [e.g., [46,47]]. Accordingly, it seems appropriate to test the Gondwanan hypothesis of ariine diversification in a chronological framework.

The final separation of South America and Africa is dated to be as recent as 105 mya [53,54], Madagascar and India separated at about 95-84 mya [51,55,56], and Australia and Antarctica initiated rifting 95 mya but remained connected until at least 35 mya [57]. While the temporal context for the separation of India-Madagascar from the major Gondwanan landmass is more controversial [56,58], it has been suggested that the Kerguelen Plateau served as a corridor allowing exchange of terrestrial and freshwater faunas between India-Madagascar and Australia via Antarctica until about 80 mya [48,51,58]. Before the Eocene, Antarctica had a temperate climate and included fish fauna found at lower latitudes today [59], such as catfishes reported from the Eocene/Oligocene boundary [1]. Likewise, after the fragmentation of Gondwana, shallow marine connections between major landmasses might have remained open until ~80 mya (e.g., Africa and South America at 84 mya [53]) or later permitting exchange of continentally-restricted marine taxa such as ariines.

Under the Gondwanan model a Mesozoic origin of major ariine clades is predicted. Previous studies addressing the timing of catfish diversification provide no support for this scenario. Lundberg et al. [10] inferred nodal ages for siluriform families using nuclear *rag1 *and *rag2 *sequences and seven fossil calibration points via Bayesian relaxed clocks (BRC) and penalized likelihood analyses. Their divergence time estimation for arioids and ariines is ~73 my and ~20 my, respectively [[10]: Fig. 2]. It is remarkable, however, that Lundberg et al. arbitrarily selected 144 my (informed by the fossil record of actinopterygians) as the maximum age constraint for the split of gymnotiforms and siluriforms. While addressing the timing of galeichthyine intercontinental disjunction, Betancur-R. and Armbruster [14] inferred 94-74 my (95% credibility interval 104-69 my) for the arioid split and 40 my (95% credibility interval 59-29 my) for the ariine split based on mitochondrial sequences and three calibration points. Instead of fixing the root of the tree to an arbitrary age, they used minimum and maximum age constraints on a terminal node based on the rising of the Panama isthmus 3.1-2.8 mya. In order to provide more accurate estimates on the divergence time of basal nodes in the arioid tree, this study selected 18 calibration points on two deep osteichthyan nodes, six non-arioid catfish nodes, and five arioid nodes (Additional file 3).

Divergence times for selected nodes derived from uncorrelated lognormal (UCLN) and BRC analyses are shown in Table 3. Both methods provided divergent estimations on the temporal context of ariine diversification. The BRC analyses estimated much older (~twofold greater) divergence times for most nodes than UCLN model. For instance, according to BRC analyses, the Ariinae stem group originated 104.8-63.9 mya (95% credibility intervals 162.2-50.0 my) and the split between New World and Old World ariines occurred 80.6-49.4 mya (95% credibility intervals 136.4-45.7 my); the UCLN model inferred 50.6-41.4 my (95% highest posterior density limits 69.8-33.4 my) and 37.8-35.1 my (95% highest posterior density limits 51.5-26.4 my), respectively (Table 3). The dates obtained with UCLN model, although most similar to those reported in previous studies [10,14], offer little support for a Gondwanan origin of ariines. Conversely, the divergence times inferred using BRC are within the range of those predicted under the vicariant model (Table 3). Similar discrepancies on the timing of cichlid divergence in the context of Gondwanan vicariance are reported in recent molecular studies. Based on molecular clock analyses, Vences et al. [60] inferred a Cenozoic origin for cichlids (consistent with the fossil record) suggesting recent transatlantic dispersal from Africa to South America. In contrast, Azuma et al. [61] inferred divergence times for cichlids that are consistent with a Gondwanan origin during the Cretaceous [see also [62]]. While the latter hypothesis is more widely accepted [e.g., [49,62], but see [63]], interestingly, different calibrations and methods have inferred ages for cichlids that are two- to three-fold different.

**Table 3:** Divergence time estimates for selected nodes.

		**BRC (my)**		**UCLN (my)**	
Taxon/Clade	Node	Complete dataset	Reduced dataset	Complete dataset	Reduced dataset
Osteicthyes		432.8 (448.8-417.1)	431.8 (448.8-416.8)	450 (450.1-449.0)	450 (450.1-449.0)
Actinopterans		405.6 (431.2-392.5)	417.1 (442.5-394.1)	395.6 (407.1-391.1)	396.3 (408.5-391.1)
Gymnotiformes-Siluriformes		229.9 (274.8-186.9)	313.2 (401.5-219.0)	196.6 (238.6-157.8)	228.0 (272.3-181.9)
Arioidea		111.8 (140.9-86.9)	156.4 (228.5-100.6)	77.0 (88.5-70.4)	89.1 (113.3-70.4)
Ariidae		88.4 (111.9-68.9)	130.7 (196.4-81.3)	57.4 (69.4-45.7)	67.1 (90.4-47.5)
Galeichthyinae		33.5 (51.9-19.9)	53.2 (102.7-21.0)	14.7 (21.0-8.9)	21.6 (38.2-8.7)
*Galeichthys peruvianus + G. ater*/*G. feliceps*		18.8 (32.5-9.6)	23.7 (56.3-6.13)	7.4 (11.4-3.9)	7.9 (16.0-2.1)
Ariinae	A	63.9 (80.9-50.0)	104.8 (162.2-63.0)	41.4 (50.0-33.4)	51.8 (69.8-36.2)
Some New World + Old World	E	58.8 (75.3-45.7)	86.7 (136.4-50.8)	35.1 (42.6-28.2)	37.8 (51.5-26.4)
Old World Ariinae	I	49.4 (64.1-37.7)	80.6 (127.8-46.5)	29.3 (35.7-23.4)	32.6 (43.9-22.5)
Indo-Pacific Ariinae (split India, Madagascar, and Asutralia-N. Guinea)	J	47.0 (61.2-35.6)	76.3 (121.1-44.1)	27.4 (33.4-21.9)	29.9 (40.3-20.6)
India-Southeast Asia		42.5 (56.1-31.8)	71.9 (115.3-40.9)	25.1 (30.1-19.5)	23.4 (32.1-15.5)
Australia-N. Guinea	O	36.9 (49.2-27.4)	59.4 (98.2-32.2)	19.3 (24.8-14.8)	20.4 (29.3-12.3)

Values in parenthesis indicate 95% credibility intervals for Bayesian relaxed clock (BRC) and 95% highest posterior density limits for uncorrelated lognormal (UCLN) model. Complete dataset: *cyt b*, *ATPase 8/6*, and *rag2 *(2934 bp). Reduced dataset: excluding *ATPase 8 *and third codon positions of *cyt b *and *ATPase 6 *(2173 bp). Nodes refer to clades in Figure 3.

While cross-validation procedures for the nine fossil-based nodes suggest no internal conflicts, several calibration points are outside the arioid tree and thus may not be much informative (Additional file 3). It is also noteworthy that, following the recommendations outlined in Lundberg et al. [10], minimum age constraints based on fossils were applied one node below the pertinent taxon (e.g., ariid fossils of Late Campanian-Early Maastrichtian were assigned to the split of ariids and anchariids). Although conservative, this procedure may have resulted in underestimated nodal ages (at least for UCLN dates). An alternative solution for improving accuracy on divergence time estimates of basal arioid nodes could come from assigning additional ariid fossils to particular subclades. However, such work requires an exhaustive morphological examination of both fossil and recent taxa. Among catfish families, ariids include the oldest and most abundant elements in the fossil record. Ariid fossils have been identified from bony (48 localities) and otolith (68 localities) remains [7] and the fragments are widespread, including records from North and South America, Europe, Asia, and Africa. The oldest ariid fossils date from the Late Campanian-Early Maastrichtian in North and South America. Old World fossil remains also include Maastrichtian otoliths assigned to the Ariidae, but the oldest bony elements are from the lowermost Eocene (Europe, Africa and Asia) [7,64]. Considering that the stratigraphic record indicates that by the Early Maastrichtian (~70 mya) ariids were already widely distributed [see [7]: Fig. seventeen part 2], the origin of the group might be older, implying a failure of preservation and/or detection of ancient fossils. If that is the case, a precise assignment of the oldest known specimens to particular ariid subclades might provide more accurate molecular clock estimations in support of the vicariant scenario.

## Conclusion

This study utilized mitochondrial, nuclear, and combined (up to ~4 kb) sequence data to infer phylogenies for arioids based on the most comprehensive taxon sampling to date (124 species/entities). While the reconstructions support the monophyly of basal groups (Arioidea, Ariidae, Galeichthyinae, and Ariinae), up to ten ariine genera (out of 25–30) validated by previous morphological studies are incongruent with the molecular phylogenies. These results stress the need for re-assessment of ariid classification.

The topologies recovered New World ariines as paraphyletic and Old World species were grouped into a well-supported clade. In further disagreement with morphological hypotheses that follow no biogeographic patterns, the molecular phylogenies group inshore ariine species into well-defined clades restricted to particular Gondwanan provinces (New World [three to five basal clades], Africa, Madagascar, India-SE Asia, and Australia-New Guinea). The general area cladogram derived from the area cladograms of cichlids, aplocheiloid killifishes, rainbowfishes, and ariines is largely congruent with the temporal sequence of events during the fragmentation of Gondwana. Nonetheless, the results obtained using BRC and UCLN methods are too variable, which hinders drawing definitive conclusions on the timing of ariine diversification in the context of Gondwanan vicariance. Further examination of additional ariid fossils might provide better calibration points for more accurate molecular clock estimates.

## Methods

### Taxon sampling

Generic nomenclature for New World and Old World ariids follows Betancur-R. et al. [19] and Marceniuk and Menezes [21], respectively. The datasets included a total of 123 ariid species/entities (see below), representing 28 genera and the two subfamilies (Galeichthyinae and Ariinae; ~230 total individuals sequenced). Based on current check lists and classifications [19,21], all but one ariid genus (*Batrachocephalus*) was examined and at least 37 species in 16 genera were not examined due to unavailability of tissue samples. The phylogenetic sampling includes ariid species from 26 countries and all major biogeographic provinces. One species of the sister family Anchariidae (*Gogo arcuatus*) was also included in the ingroup (Arioidea sensu [6]); three distantly-related catfish families were used as outgroups (Heptapteridae, Ictaluridae, and Auchenipteridae, listed in [19]). Additional outgroups were also selected for divergence time estimations (see below). Material examined is listed in Additional file 1. Institutional abbreviations are as listed at [65].

For several taxa, two or more individuals were sequenced for at least one gene region (mostly *ATP synthase 8/6*). As many as 15 species revealed significant morphological and/or molecular (>1.5% genetic divergence) differentiation among localities, suggesting that several potential undescribed/unrecognized species exist. In such cases, different entities were analyzed as separate terminals, referring the individuals collected close to the type locality (if known) as the nominal species and the allopatric entities as *affinis *(aff.). Undescribed or unidentified species were listed to the genus level (sp.), whereas dubious identifications were treated as *confer *(cf.). Old World species placed in *Sciades *by Marceniuk and Menezes [21] were referred as '*Sciades*' (see Discussion).

### DNA data and phylogenetic reconstructions

Targeted mitochondrial regions included partial *cytochrome b *(*cyt b*) and complete *ATP synthase *subunits 8 and 6 (*ATPase 8/6*) protein-coding genes, and partial *12S *and *16S *ribosomal genes. Nuclear evidence included partial *recombination activating gene 2 *(*rag2*). Laboratory protocols, PCR conditions, utilized primers, and sequence alignment procedures are as described in Betancur-R. et al. [19]. Two additional *rag2 *primers were designed for several ariid taxa that failed to amplify using the combination MHRAG2-F1 and MHRAG2-R1 [19,66]: rag2.Ari.F, 5'-GAGCCTCACAGTGAAAACCCYGAG-3'; rag2.Ari.R, 5'-CTCCCTCTCCATCACTGCTGTAC-3'. Different combinations of these four primers yielded successful amplifications in all cases.

Phylogenetic reconstructions were performed under MP, ML, and BI criteria. The initial conditions used for different analyses are summarized in Table 1. The MP reconstructions were conducted in PAUP* v. 4.0b10 [67] via heuristic searches with random addition (RA) of sequences and tree-bisection-reconnection (TBR); clade support was evaluated using non-parametric bootstrapping with RA and TBR. For ML and BI, the best-fit models of sequence evolution were estimated using the Akaike information criterion (AIC) in ModelTest v. 3.7 [68]. All analyses were run unpartitioned. The ML analyses were performed in the programs Garli v. 0.951/0.96 [69] and RAxML v.7.04 [70]. Garli searches were conducted using automatic termination (genthreshfortopoterm command). RAxML searches were run in the CIPRES portal v. 1.13 [71] under default configurations. ML nodal support was evaluated in RAxML using the rapid bootstrapping algorithm with automatic estimation of runs. For both Garli and RAxML searches, several runs from random-starting seeds were performed to check convergence of likelihood scores. Model parameters were estimated simultaneously (i.e., unfixed). Remaining settings were left at their default values.

The BI analyses were performed in MrBayes v. 3.1.2 [72] via Markov chain Monte Carlo (MCMC) iterations. The MCMC analyses were conducted in triplicate using four chains and sampling trees every 100 generations. Conservatively, 25% of the first trees sampled in each MCMC run were discarded as burn-in. Marginal probabilities of summary parameters, consensus phylograms, and posterior probabilities of nodes were estimated from the post-burn-in samples of the three independent runs combined (Table 1). To confirm that post-burn-in trees were sampled from the actual MCMC posterior distribution, marginal parameters (MrBayes log file) were analyzed using the Effective Sample Size (ESS) statistic in the program Tracer [73]; ESS greater than 200 suggests that MCMC searches were run long enough to accurately represent the posterior distribution [73].

### Hypothesis testing

Alternative hypotheses were compared to the molecular topologies using the parsimony-based nonparametric Templeton test and the likelihood-based Shimodaira-Hasegawa (SH) test [74] as implemented in PAUP*. Three major hypotheses were tested: (1) Kailola's [15] morphological phylogeny (Figure 2A), (2) recent generic classifications derived from anatomical data [15,21], and (3) a Gondwanan vicariance model for ariines. A MP tree that represented a particular hypothesis was estimated using constrained tree searches in PAUP*. The constrained trees were compared to the unconstrained MP topologies using the Templeton test. Likewise, constrained and unconstrained topologies were estimated under ML in Garli and compared via SH tests (1000 replicates and RELL sampling). For ML comparisons, the best-fit model and parameters were selected using the AIC.

### Biogeographic inferences

Ancestral areas were reconstructed via dispersal-vicariance analyses as implemented in the program DIVA v. 1.2 [75]. DIVA analyses were performed for inferring: (1) 'center of origin' for New World ariines (unit areas coded as South America/southern Central America, Mesoamerica/North America, Old World); (2) ancestral distribution of basal ariine lineages and widespread Indo-Pacific taxa (unit areas coded as New World, Africa, Madagascar, India-SE Asia, Australia-New Guinea). The number of ancestral areas was restricted to two using the maxareas command. All DIVA analyses were run on MP and ML topologies.

An area cladogram was constructed by replacing the names of terminal taxa with their distributions. The area cladograms for the Ariinae were compared to those inferred by Sparks and Smith [48] for cichlids, aplocheiloid killifishes, and rainbowfishes (Melanotaenioidei). A general area cladogram based on the four fish groups was then derived and compared to the general area cladogram hypothesized by Sparks and Smith using the latter three groups only. To handle widespread taxa, redundant distributions, and missing areas, a component analysis [76-78] was performed in the program Component v. 2.0 [79,80]. Component analyses were run using the nearest-neighbor interchanges algorithm by minimizing the number of leaves added. Host without associates (= missing areas) were treated as missing information. Widespread associates were dealt under assumption 2 (i.e., areas including widespread taxa have monophyletic, paraphyletic, or polyphyletic relationships [76-78,81]) by arbitrarily removing all but one area from the distribution of each widespread taxon [79,80]. The general fish area cladograms were further compared to the geological area cladogram of Gondwanan breakup (summarized by Sparks and Smith [48] based on Smith et al. [55] and Storey [56]).

### Divergence time estimations

Relative rate tests based on likelihood were performed on eight clades with different nesting hierarchies using the software r8s v. 1.71 [82,83]. Four out of the eight comparisons suggested significant departures from a clock-like behavior (*p *< 0.05). Thus, two different methods that do not assume a strict molecular clock were used for chronological estimations, BRC as implemented in Multidivtime [84] and UCLN as implemented in Beast v. 1.48 [73,85]. BRC analyses were run unpartitioned with detailed procedures outlined in Betancur-R. and Armbruster [14]. The initial tree was estimated using ML (Garli) on combined protein-coding sequences (mitochondrial *cyt b *and *ATPase 8/6*, and nuclear *rag2*). In addition to the combined dataset, based on the recommendations outlined in Hurley et al. [86], divergence times were also estimated excluding the entire *ATPase 8 *fragment as well as third codon positions of *cyt b *and *ATPase 6 *to reduce the effect of saturated mitochondrial sequences [see also [87]]. Eighteen calibration points on two deep osteichthyan nodes, six non-arioid catfish nodes, and five arioid nodes were set as maximum (six points only) and minimum age constraints based on median fossil ages (see Additional file 3; material examined is listed in Additional file 1 [after [7,10,14,33,61,86,88-93]]). To assess internal consistency among calibration points, fossil-based cross-validation procedures were performed in r8s under penalized likelihood (PL) using truncated Newton algorithm and fossilconstrained command [94]. Priors for the BRC analyses were calibrated as follows: rttm, rttmsd, and bigtime parameters were set to 45.0 (= 450 my; estimated divergence time between sarcopterygians and actinopterygians [61,88]); rtrate and rtratesd parameters were both set to the mean value for the total evolution of all branches from the root to the tip of the tree divided by rttm (rtrate and rtratesd = 0.0468, combined dataset; rtrate and rtratesd = 0.0603, reduced dataset). Other priors were set to their default values and/or as specified in Betancur-R. and Armbruster [14].

For UCLN analyses, the initial Beast file was generated in BEAUti [73]. The substitution model was GTR+I+Γ (as selected by AIC) with base frequencies estimated empirically, using two data partitions (first and second codon positions combined and third codon positions) for the complete dataset and three partitions (separate codon positions) for the reduced dataset. All parameters were unlinked and mean substitution rate was unfixed. A starting chronogram that replaced the UPGMA default tree and satisfied all UCLN priors was generated under PL in r8s. The tree prior parameter selected was Yule process, which assumes a constant speciation rate per linage and is more appropriate for species-level phylogenies [73]. Calibration nodes were constrained using lognormal-distribution priors whenever there was confidence that fossils are close in time to cladogenesis (i.e., the stratigraphic record is fairly complete [95]; e.g., Osteichthyes and Actinoperi [61,86,88,89]). As inferred by molecular clock analyses, the origin of siluriforms (i.e., 175-130 mya [8-10]) is much older than predicted by the oldest fossil (ca. 68-73 mya), suggesting a gap in the stratigraphic record. Thus, catfish nodes were constrained under uniform-distributions (Additional file 3). Tree root was set to 450 using uniform distribution (similar to bigtime parameter in BRC). Other priors and operators were set to their default values. The MCMC analyses were run in duplicate for 3 × 10^7 ^generations, sampling trees every 1000 generations. The MCMC log files were combined in Tracer to summarize posterior divergence times with 95% highest posterior density limits; ESS values greater than 200 were reached for all marginal parameters after discarding 20% of the first trees as burn-in.

## Authors' contributions

RBR conceived the study, collected data, made the analyses, and drafted the manuscript.

## Supplementary Material

Additional file 1 **Material examined and GenBank Accession numbers**. The material listed indicates origin of samples, collection information, and GenBank accession numbers for each gene region. Two letter country codes follow ISO-3166. NV, not vouchered; NCA, not catalogued. Click here for file

Additional file 2 **Congruence among reconstruction methods and data partitions**. For each analysis filled and open cells indicate presence or absence of a particular clade, respectively. Numbers in cells indicate nodal support (whenever available, see Table 1), with bolded values for MP or ML bootstrap ≥ 75% and BI posterior probabilities ≥ 0.95. Nodes refer to clades in Figure 3. Click here for file

Additional file 3 **Calibration points used for divergence time estimations**. Nodal constraints were set based on MRCA's (most recent common ancestor) according to the topologies presented in Azuma [[61]: Fig. 2, basal fish nodes], Lundberg [[10]: Fig. 2, siluriforms], Hardman [[33]: Fig. one, pimelodids], and Figure 3 (arioids). BRC, Bayesian relaxed clock; UCLN, uncorrelated lognormal model; U, maximum age constraint; L, minimum age constraint. Click here for file
